# I Know It When I See It: The Challenges of Addressing Corruption in Health Systems: Comment on "We Need to Talk About Corruption in Health Systems"

**DOI:** 10.15171/ijhpm.2019.48

**Published:** 2019-07-07

**Authors:** Jillian Clare Kohler

**Affiliations:** WHO Collaborating Center for Governance, Accountability and Transparency in the Pharmaceutical Sector and Leslie Dan Faculty of Pharmacy, University of Toronto, Toronto, ON, Canada.

**Keywords:** Corruption and Health Systems, Health Policy, Health System Governance

## Abstract

In this commentary, I argue that corruption in health systems is a critical and legitimate area for research in order to strengthen health policy goals. This rationale is based partly on citizen demand for more accountable and transparent health systems, along with the fact that the poor and vulnerable suffer the most from the presence of corruption in health systems. What is more, there is a growing body of literature on the impact of corruption in the health system and best practices in terms of anti-corruption, transparency and accountability (ACTA) strategies and tactics within the health system. Still, we need to support ACTA integration into the health system by having a common definition of corruption that is meaningful for health systems and ensure that ACTA strategies and tactics are transparent themselves. The 2019 Consultation on a proposed Global Network on ACTA in Health Systems is promising for these efforts.

In his concurring opinion in Jacobellis v. Ohio (1964), then United States Supreme Court Justice, Potter Stewart, stated “*I know it when I see it*” with regards to what is hard core pornography. When identifying corruption, there is often the risk of adopting the same nebulous approach, as corruption is often not well defined, or may have different meanings depending on the cultural and institutional context.^1^ In their commentary on “*We Need to Talk About Corruption in Health Systems*” Hutchinson et al^2^ identify and explain five leading reasons why corruption in health systems has not been confronted well. In this commentary, I examine each of them.

First, the authors importantly identify key barriers to eradicating corruption from health systems. Corruption in health systems potentially can undercut national public health goals, as well as global health goals, such as Sustainable Development Goal (SDG) #3: *Good Health and Well-Being*.^3^ As Mackey et al^4^ have argued, combating corruption should be a core value of the SDGs, given its linkages with human rights, equity, economic development and Universal Health Coverage (p. 640).

The authors underscore that we need to initiate (what I assume is) a public policy-driven conversation about corruption in health system. This conversation *is happening*. To provide some context, as far back as 1996, then World Bank President James D. Wolfensohn expressed that a ‘cancer of corruption’ was depleting resources from the world’s poor in his speech at the Annual Joint Meeting of the International Monetary Fund and the World Bank.^5^ Wolfensohn’s high profile speech helped initiate a growing exploration of the impact of corruption in development financing and operations, including the health sector.^6-8^

The health system is indeed a rich place for the study of corruption. As a result of its fragmentation, technical complexity, and the vast sums of money involved in the purchase of health products and its operations, it is an optimal space for corruption to thrive. Corruption is found throughout the health system; from petty corruption at the health facility level to corruption that takes place at the highest level of the state.^8^

There is indeed a growing body of evidence which is documenting the pernicious effect corruption has had on health systems, particularly in terms of health equity goals. For instance, Vian’s research on corruption and its consequences for public health, the European Commission’s *Study on Corruption in the Healthcare Sector,* and Transparency International’s recent study on corruption risks in health system delivery.^9-11^ Transparency International has in fact focused its policy work on corruption in the health sector as far back as in 2006 as documented in its annual publication, *The Global Corruption Report*.^12^

A number of United Nations organizations, including the World Bank, the World Health Organization (WHO), the Global Fund, and the United Nations Development Programme (UNDP) have also addressed corruption through their institutional policy and country operational work. Still, anti-corruption efforts by international organizations have regularly been obfuscated by more benign concepts, such as good governance, risk management, and health systems strengthening.^13-15^ Anti-corruption, transparency and accountability (ACTA) efforts, surely need to be identified as such in order to make progress towards the reduction of corruption in the health system. Hutchinson et al illuminate other key barriers; I examine each in the below.

## Reason 1: it is difficult to define corruption.

How *do* we define corruption? It is undeniably a thorny enterprise to arrive at a single definition of corruption given its complexity and reach. Hutchinson et al point out that corruption in its many forms may not be considered as such uniformly, and they provide examples of why this is the case. What *is* corruption (and here I am limiting it to its manifestations in the healthcare system) may very much depend on its health systems institutional context and cultural setting. Certainly, gifts to physicians in some countries may be considered as “gratitude payments.”^16-22^ This may not be viewed as corruption *per se* but as a culturally acceptable practice – often related to the poor salaries that health professionals receive.^23-26^

But here is where we must draw a line; we need to stop making allowances for the vast smorgasbord of definitions that populate the field. Hutchinson et al emphasize that it is difficult to define corruption. For this reason, we need a *common definition* that translates easily into practice and across geographical, social, institutional and cultural terrain and is relevant for the health system. Transparency International has an expansive definition of corruption that could serve as the common definition. It is “Corruption is the abuse of entrusted power for private gain. It can be classified as grand, petty and political, depending on the amounts of money lost and the sector where it occurs.”^27^ The adoption of a common definition of corruption, such as the one proposed, which is meaningful for the health system, could potentially be advanced within a global health forum, such as the World Health Assembly.

## Reason Two: Practices that seem corrupt may actually be seen by those concerned, both providers of services and their beneficiaries, as the only means of enabling some fragile health systems simply to keep working. 

To be sure, informal payments to health professionals may be viewed as vital for the functioning of a poorly resourced public healthcare system,^28^ and cultural factors.^24,29-31^ For instance, Cockcroft et al,^32^ in their study of unofficial payments in the health sector of Baltic states, found that the majority of the patients they surveyed – nearly 50% – did not consider unofficial payments to health workers as corruption. While informal payments may help healthcare services in some cases, they are undeniably potential barriers to healthcare services for the poor and vulnerable. Accordingly, we need to stop making allowances for corruption, even when it may result in socially desirable results.

## Reason Three: How do we conduct research on corruption in ways that capture what is really happening?

Researching corruption, whether it is in the health system, or elsewhere, is challenging given that corruption tends to be hidden. Very often stakeholders are reluctant to speak about corruption openly; they may be committing corrupt acts themselves or fear reprisals by those who are committing it. Hutchinson et al underscore that research efforts may not capture the truth of corruption and that researchers face the moral quandary of seeking access to those who may very well be engaged in exploitative behaviour in order to report it. These concerns are certainly valid. Still, researchers are doing a vital part in anti-corruption efforts by seeking to identify the how, why, and what of corruption in the health system even when research relies on perceptions.^33^ And while research may not illuminate the full story of how corruption is taking place and detail the granularity of its impact, even exploratory research is absolutely fundamental to grasping what strategies and tactics will work most effectively to reduce the risk of corruption in health systems.

## Reason Four: Is it even legitimate to study corruption?

Citizen demand for more accountable and transparent health systems has in fact helped promote more policy attention to corruption generally and in health systems. What is certain is that evidence-based research can help promote sound public policy. Corruption within the health system harms the poor and most vulnerable population groups the most.^34^ For instance, a 2015 study by Transparency International on corruption in Africa reported that the poor are often forced to pay bribes for access to “free” public services.^35^ When considering the need for governments to respond to citizen demands and the impact of corruption in the health system on the poor and vulnerable, it is indeed a valuable enterprise to study corruption.

## Reason Five: We know too little about how to tackle corruption. 

Hutchinson et al cite a recent Cochrane review that did not find any studies with empirical evidence concerning the effects of strategies to reduce corruption. What is more, the review found that only a small number of the studies could be used to inform further evaluations.^36^ Still, there is an ever growing body of literature that is examining the effect of ACTA measures that is informative and often helpful in terms of identifying best practices.^37^ While we need to know more about what are the optimal strategies and tactics to tackle corruption, we are certainly gaining knowledge about what works well and where.

And as a final consideration, what do we do about corruption in the health sector? The authors recommend convening stakeholders in the health system that can then speak openly about corruption and reach agreement on realistic priority areas and actions. This has recently come to fruition: the WHO, UNDP, and the Global Fund in February 2019 in Geneva convened a multi-stakeholder consultation on a proposed Global Network on ACTA in Health Systems.^38^ This Consultation convened over one hundred policy-makers, researchers, non-governmental organizations, government officials and representatives from United Nations organizations to discuss corruption in the health system. The Consultation concluded with a proposed workplan for the Network. It included outputs such as: (1) rationalizing internal control and assurance models in health systems using fraud and corruption risk assessment methodologies; (2) monitoring and evaluation of ACTA measures for health; (3) capacity development on ACTA in the health sector for multiple stakeholders; and (4) the integration of ACTA into health systems strengthening normative guidance.

In conclusion, it is helpful to consider what are the potential barriers to addressing corruption in health systems as this can assist researchers and policy-makers to overcome them. ACTA work clearly needs to be more fully integrated into health system policy and practice. For certain, global initiatives are underway and progress in the fight against corruption in the health system appear promising. “Corruption is deeply embedded in social, political and economic dynamics, and cannot be isolated from them because of its many interconnected causes and effects.”^39^ Given this inherent complexity, we need to learn more about how to address corruption in health systems. For this, we need to continue the conversation about corruption and determine how to best overcome the barriers Hutchinson et al advance.

## Ethical issues

Not applicable.

## Competing interests

JCK was a consultant to the WHO for the convening of the Proposed Global Network on ACTA in Health Systems.

## Author’s contribution

JCK is the single author of the paper.
